# Supplementary material for: Asymmetric Synthesis of Quaternary Hydantoins via a Palladium-Catalyzed Aza-Heck Cyclization
Source: J Am Chem Soc. 2025 Nov 14;147(49):44692–8. doi: 10.1021/jacs.5c16022 (PMC12703750; doi:10.1021/jacs.5c16022)

D:\data\2020\DWatson_Idowu_TDI01052

DWatson_Idowu_TDI01052 #47-74 RT: 0.32-0.46 AV: 28 NL: 9.01E8
T: FTMS + p ESI Full ms [133.5000-2000.0000]

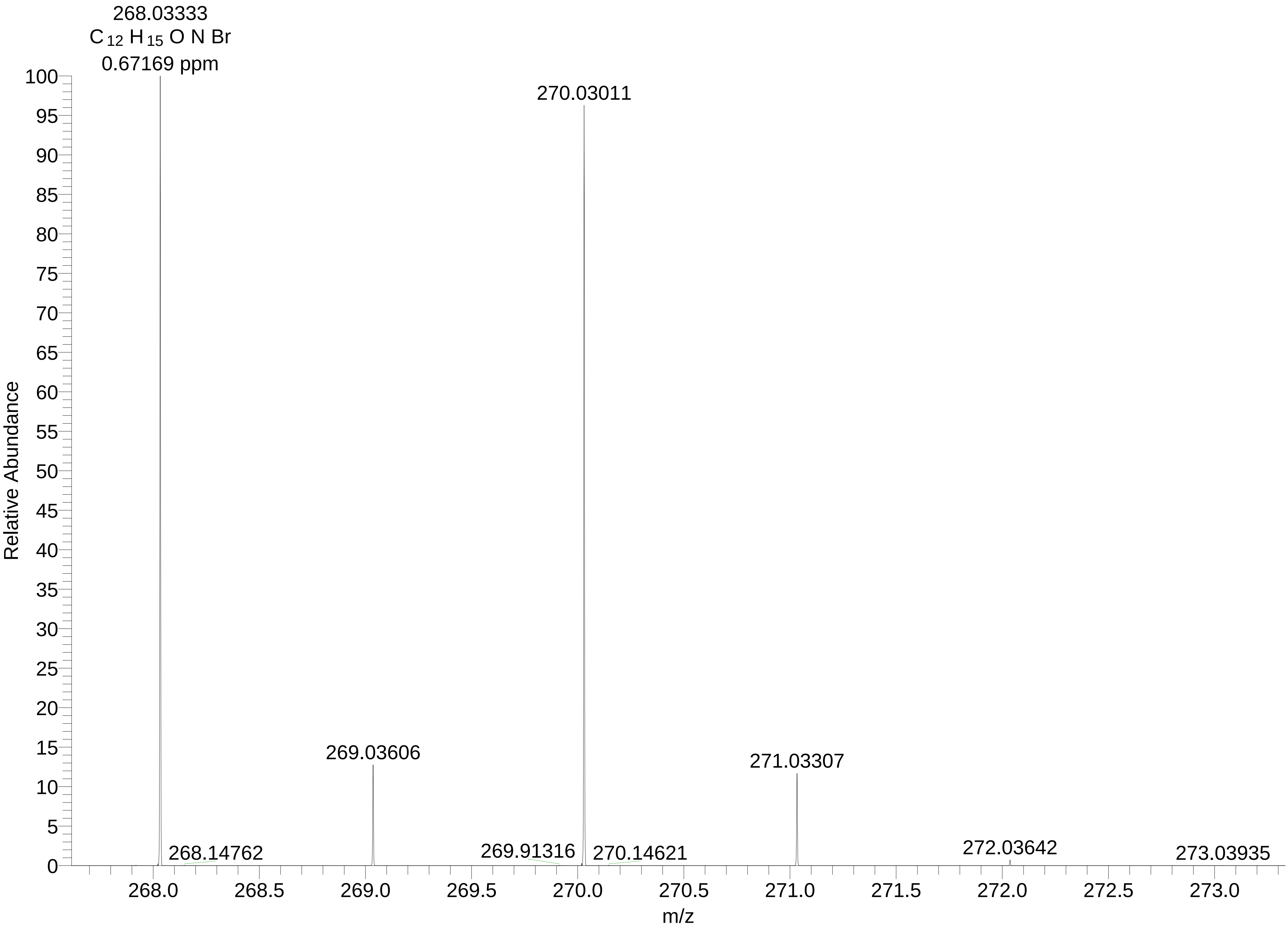

Supplement: Supplementary file 2 [file ja5c16022_si_002.zip › All NMR FID Files/S20/S20_AllNMR/HRMS/DWatson_Idowu_TDI01052.pdf]
